# Assessing agonistic potential of a candidate therapeutic anti-IL21R antibody

**DOI:** 10.1186/1479-5876-8-50

**Published:** 2010-05-26

**Authors:** Yongjing Guo, Andrew A Hill, Renee C Ramsey, Frederick W Immermann, Christopher Corcoran, Deborah Young, Edward R LaVallie, Mark Ryan, Theresa Bechard, Richard Pfeifer, Garvin Warner, Marcia Bologna, Laird Bloom, Margot O'Toole

**Affiliations:** 1 Pfizer, Global Biotherapeutic Technologies, Cambridge, MA, USA; 2 Pfizer, BioTherapeutics Clinical Statistics, Pearl River, NY, USA; 3 Pfizer, Inflammation and Immunology, Cambridge, MA, USA; 4 Pfizer, Drug Safety Research and Development, Chazy, NY, USA; 5 Shire Pharmaceuticals, Lexington, MA, USA; 6 Pfizer, Drug Safety Research and Development, Andover, MA, USA; 7 Pfizer, BioTherapeutics Development and Strategic Operations, Cambridge, MA, USA; 8 Pfizer, BioTherapeutics Clinical Translational Medicine, Cambridge, MA, USA; 9 35 Cambridge Park Drive, Cambridge, MA 01240, USA

## Abstract

**Background:**

Selective neutralization of the IL21/IL21R signaling pathway is a promising approach for the treatment of a variety of autoimmune diseases. Ab-01 is a human neutralizing anti-IL21R antibody. In order to ensure that the activities of Ab-01 are restricted to neutralization even under *in vitro *cross-linking and *in vivo *conditions, a comprehensive assessment of agonistic potential of Ab-01 was undertaken.

**Methods:**

*In vitro *antibody cross-linking and cell culture protocols reported for studies with a human agonistic antibody, TGN1412, were followed for Ab-01. rhIL21, the agonist ligand of the targeted receptor, and cross-linked anti-CD28 were used as positive controls for signal transduction. *In vivo *agonistic potential of Ab-01 was assessed by measuring expression levels of cytokine storm-associated and IL21 pathway genes in blood of cynomolgus monkeys before and after IV administration of Ab-01.

**Results:**

Using a comprehensive set of assays that detected multiple activation signals in the presence of the positive control agonists, *in vitro *Ab-01-dependent activation was not detected in either PBMCs or the rhIL21-responsive cell line Daudi. Furthermore, no difference in gene expression levels was detected in blood before and after *in vivo *Ab-01 dosing of cynomolgus monkeys.

**Conclusions:**

Despite efforts to intentionally force an agonistic signal from Ab-01, none could be detected.

## Background

IL21 (interleukin 21) is a type I cytokine produced by activated CD4+ T cells and natural killer (NK) T cells [1-4]. It promotes both B cell function [2], and growth of the TH17 T cell subset involved in chronic inflammation [5]. Involvement of the IL21 pathway has been demonstrated in a variety of pro-inflammatory and autoimmune animal models [6-10]. Inhibition of the IL21/IL21R pathway therefore represents a promising therapeutic strategy for treatment of chronic inflammatory and autoimmune conditions [11]. We have taken the approach of blocking IL21-mediated activation by developing Ab-01, an antibody that selectively binds the high-affinity alpha chain of the IL21 receptor, IL21R. Ab-01 blocks the binding of IL21 to IL21R and inhibits IL21-mediated activation [12].

Ab-01 was selected based on the property of inhibiting (antagonizing) rhIL21-mediated cell activation [13], (Arai *et al*. Journal of Translational Medicine, in press). Given that the therapeutic goal of Ab-01 is the down-modulation of autoimmune disease activity, it was important to ensure that Ab-01 cannot deliver an activation signal, even when cross-linked. Since Ab-01 is an antagonistic antibody, we did not expect that agonistic activity would be detected. However due diligence dictated that all efforts should be made to force an agonistic signal so that potential risks and biomarkers associated with any agonistic activity could be thoroughly assessed and understood. The need for such due diligence was highlighted by the clinical experience with TGN1412, an anti-CD28 candidate therapeutic antibody that, in stark contrast to Ab-01, was developed based on immune system activating activity [14]. Immunotoxicity had not been observed in preclinical studies done in rhesus monkeys, but within hours of clinical administration, life-threatening organ failure associated with cytokine storm was observed in all treated volunteers [15]. So even though Ab-01 is an immune system antagonist and TGN1412 an immune system agonist, we conducted an in-depth search for activation signals induced by *in vitro *cross-linked Ab-01 and examined the effects of Ab-01 when administered *in vivo *to cynomolgus monkeys.

In addition to antagonist activity of Ab-01 versus the agonist activity of TGN1412, there are other important distinctions between Ab-01 and TGN1412, particularly in the preclinical data packages of the two antibodies. Binding of TGN1412 to rhesus T cells had been demonstrated prior to clinical testing [16], but biological activity of TGN1412 on rhesus cells had not been extensively explored. In contrast, we have shown that Ab-01 blocks rhIL21-mediated cell activation in cynomolgus monkeys, the Ab-01 safety study species [13], (Arai *et al*. Journal of Translational Medicine, in press), and that the IC50 of Ab-01 in cynomolgus monkey is only about 3.5-fold higher than the IC50 in human (Arai and LaVallie, unpublished data). These studies established that Ab-01 activity is very similar in cynomolgus monkeys and in human, whereas the activity of TGN1412 in rhesus was clearly very different from the activity in humans.

The clinical experience with TGN1412 has heightened concern within the medical, regulatory and drug development communities regarding immunotoxic potential of immuno-modulatory antibody therapeutic candidates [15-19]. As a result of the review conducted in the aftermath of the experience with TGN1412, comprehensive efforts were undertaken to identify *in vitro *protocols capable of revealing the immunotoxic cytokine storm-inducing properties of TGN1412. Using purified PMBCs from healthy donors, Stebbins *et al*. found that TGN1412, when cross-linked *in vitro *using any of three methods, induced secretion of a set of cytokines that had been associated with the cytokine storm syndrome in the clinic [14]. Notably, PBMC from rhesus did not give this response to *in vitro *cross-linked TGN1412, and this *in vitro *dichotomy between humans and the safety study species mirrored the dichotomy that had been observed *in vivo*. Two lines of evidence supported the hypothesis that the *in vitro *cross-linking assay system was a relevant surrogate read-out for the observed *in vivo *immunotoxicity of TGN1412: a) there was a concordance in the cytokines induced in humans *in vivo *and *in vitro*, and b) the cytokine storm-associated response was not observed either *in vivo *or *in vitro *in rhesus.

Our strategy for *in vitro *testing of the ability of Ab-01 to induce an activation signal was to follow and significantly expand upon the cross-linking protocols reported by Stebbins *et al.*[14]. We used two types of positive controls: rhIL21 stimulation for signal transduction through the therapeutic target IL21R, and *in vitro *cross-linked anti-CD28 for induction of cytokines associated with immunotoxicity. In parallel, we identified biomarkers of rhIL21 pathway agonism in monkey blood [13], (Arai *et al. *Journal of Translational Medicine, in press), and report here on the levels of these biomarkers in blood from monkeys collected before and after high dose IV administration of Ab-01.

## Methods

### Protein reagents: rhIL21, Ab-01, anti-CD28, and control antibodies

Most protein reagents used in this study - rhIL21, anti-rhIL21 receptor antibody Ab-01, control antibody human IgG_1_α-tetanus triple mutant (IgG_1_TM), control antibody human IgG_1 _α-tetanus wildtype (IgG_1_) and control antibody human Fc control (IgGFc) - were made by the Global Biotherapeutics Technologies Department at Pfizer, Cambridge, MA. The three mutations common to the Fc portion of Ab-01 and IgG_1_TM reduced their potential effector activity. Antibodies with these mutations had undetectable activity in ADCC or C1q binding assays [20,21]. An antibody with severely compromised effector function was chosen for development because the therapeutic goal is to block the interaction of IL21 with IL21R, and therefore minimization of effector function is desirable. rhIL21 was used as a positive control for signal delivery through IL21R. Anti-CD28 antibody ANC28.1/5D10(Ancell, Bayport, MN) was used as a positive control for cell activation mediated by cross-linked antibodies. Endotoxin levels in all proteins reagents were below 1.0 EU/mg.

### Adherence and confirmation of adherence of antibodies to wells

Ab-01, anti-CD28, and control immunoglobulins (human IgG1 α-tetanus triple mutant, human IgG1 α-tetanus wild type and human Fc control) at 5 ng, 50 ng, 100 ng, 300 ng, 1 μg or 10 μg per well were presented to Daudi cells and purified human PBMC using the conditions for coating onto plastic wells as previously described [14]. For dry coating, a total volume of 50 μL containing the indicated concentration of IgG reagent was applied to each well (96-well polystyrene Corning High Bind plates, Corning, Lowell, MA). Uncovered plates were left to dry in a tissue culture hood at room temperature overnight. For the anti-IgG-coated wells, the indicated concentration of IgG reagent was added in a volume of 100 μL to wells of goat anti-human IgG plates (H+L) (BD Biosciences, Bedford, MA) at room temperature for 1 hour, and then agitated overnight at 4°C. For the wet coating method, IgG reagents at the indicated concentration were added in 100 μL in PBS (PH = 7.2) to wells of 96-well polystyrene Corning High Bind plates (Corning, Lowell, MA). Plates were agitated at room temperature for 1 hour and incubated at 4°C overnight. All antibody-coated plates were washed three times with PBS CMF (PBS free of calcium/magnesium, pH = 7.2) before addition of cells.

Following the completion of cell culture procedures described below, the persistence of bound immunoglobulin on wells was confirmed by ELISA detection of human IgG. Following cell harvest and 4 washes with 0.03% Tween-20 in PBS, 100 μL/well of 1:2,000 dilution of HRP-coupled mouse anti-human IgG (Southern Biotech, Birmingham, AL) was added. Plates were agitated gently for 30 min, washed 4 times with 0.03% Tween-20 in PBS and 100 μL/well of BioFX TMB HRP Microwell Substrate (BioFX Laboratories, Inc., Owings Mills, MD) added. Color development was allowed to proceed for 5 minutes at room temperature. The reaction was stopped with 50 μL/well 0.18N H_2_SO_4_. The relative amount of bound antibody was recorded using a SpectraMax Plus plate reader (Molecular Devices, Sunnyvale, CA). Bound IgG was confirmed by ELISA in all wells used in the studies reported here. ELISA results showed increasing IgG-specific signal between the 0.1 μg/well and 1 μg/well concentration, with no difference in signal between the 1 μg/well and 10 μg/well concentrations (data not shown).

### Human PBMC isolation and assay for effects of cross-linked antibody

Human blood was obtained from Research Blood Components, Brighton, MA. An IRB-approved consent form was obtained from each donor consenting to blood donation for research purposes. Peripheral blood mononuclear cells (PBMCs) were isolated from 136-450 mL of blood using Sodium Citrate CPT Vacutainer tubes according to the manufacturer's instructions. PBMCs were washed twice in PBS (pH = 7.2), and differential cell counts were measured using a Pentra 60C (Horiba ABX Diagnostics, Irving, CA). Cells were resuspended in RPMI with complete supplements and plated at 2.5 × 10^5 ^cells in 100 μL/well and cultured for the durations indicated. Experiments testing the effects of cross-linked Ab-01 were conducted on PBMCs from 10 different individual human donors, and positive and negative controls were performed contemporaneously.

Levels of IFNγ, IL1β, IL2, IL4, IL5, IL8, IL10, IL12p70, IL13, and TNF were determined using 10-spot 96 well MSD plates (MS6000 Human TH1/TH2 10-Plex Kit, Meso Scale Discovery, Gaithersburg, MD) according to the manufacturer's instructions. In addition, levels of IL6 and CCL3 were determined by customized 2 spot MSD 96 well plates (Meso Scale Discovery). Tests were run in triplicate and Student's t-tests were used to identify significant differences. Fold changes were calculated for each donor by dividing values of cytokine concentration from Ab-01 cross-linked groups by values from control antibody cross-linked groups. rhIL21-dependent fold change responses were calculated by dividing values in the presence of rhIL21 to media control. The rhIL21 stimulation controls were included both on plates pre-coated with anti-IgG and on plates that were not pre-coated.

In addition to measuring secreted protein levels, RNA expression levels were also measured. Following harvest of supernatants, RNA was isolated from cells by addition of 125 μL RLT lysis buffer (Qiagen, Valencia, CA) containing 1% β-mecaptoethanol. Total RNA isolation was performed using the QIA Shredder and RNeasy Mini kit kits (Qiagen, Valencia, CA) according to the manufacturer's recommendations. All of the samples were subjected to a DNase on-column treatment to remove potential DNA contamination, and then purified using the RNeasy Mini kit.

A phenol:chloroform (1:1) extraction was performed subsequently, and RNA was repurified using the RNeasy Mini kit. Eluted RNA was quantified using a ND-1000 Spectrophotometer (Nanodrop, Wilmington, DE). RNA was converted to cDNA using the Applied Biosystems High Capacity cDNA Archive kit with RNase Inhibitor at 50 U/sample (Applied Biosystems). cDNA samples were stored at -20°C pending TaqMan^® ^assay.

RNA expression levels in human PBMCs were measured by Human Immune TaqMan^® ^Arrays (Applied Biosystems) using the ABI 7900HT Sequence detector (Sequence Detector Software 2.2.3) according to manufacturer's instructions. Relative quantification (RQ) values for all data from the Human Immune TaqMan^® ^Array were calculated from ΔΔCt values [22] using the Sequence Detection System Software, and further analyzed in a Spotfire-guided application (Spotfire DecisionSite 8™, TIBCO Software Inc. Somerville, MA) developed within the Pfizer Global Biotherapeutics Technologies Bioinformatics Department. The genes used to normalize for RNA input for samples run on the Human Immune TLDA (human PBMC samples) were *GusB, TFRC *and *PGK1*. Tests for significance were performed by subjecting ΔΔCt values for each detector to one-way ANOVA analysis with respect to time and culture condition. The Benjamini-Hochberg (BH) - corrected p-value (FDR - False Discovery Rate) was calculated to adjust for multiplicity of testing [23]. Each sample measured by TLDA was assayed once. Three factors supported the decision to run a single TLDA per sample: 1) extensive experience has shown extremely small TLDA technical variability, 2) at least 5 biological replicates were tested for each data point, and 3) the volume of blood (400 mL) permitted by the blood donor program usually did not yield sufficient RNA for duplicates.

### Test for gene activation in blood from cynomolgus monkeys treated with Ab-01

Adult male cynomolgus monkeys (*Macaca fascicularis*; Covance Research Products, Inc., Alice, TX) weighing 3.0 to 5.6 kg were singly housed and cared for according to the American Association for Accreditation of Laboratory Animal Care guidelines. The internal Institutional Animal Care and Use Committee approved all aspects of this study. FACS analysis was performed to compare the binding of Ab-01 to PBMCs from human and monkey, (see Additional file 1, Figure S1). Animals were administered a single 100 mg/kg dose of Ab-01 by means of bolus intravenous infusion via saphenous vein catheter (22G 1" Surflo, Terumo Co, Somerset, NJ). Control monkeys received no treatment. Blood samples were collected from control monkeys at the indicated time points over the course of 56 days. Pre-dose blood samples were obtained from each of two treated monkeys. Post-dose samples were collected from each of three treated monkeys at the time points indicated in Additional file 2, Table S1. Post-dose blood samples from two treated monkeys were collected at 6 hours, and at 2 weeks, and from 3 monkeys at 1 day. Immediately upon collection, blood (1 mL) was added to tubes containing sodium citrate [0.1 M], inverted and then centrifuged at 1200 × g for 5 minutes to pellet cells. The plasma was removed and 500 μL of RPMI 1640 added to the blood pellet, 2.6 mL of RNAlater (Ambion, Austin, TX) added and handled in accordance with manufacturer's instructions.

RNA was isolated using the Human RiboPure™-Blood Protocol (Ambion). Cells were lysed in a guanidinium-based solution and initial purification of the RNA by phenol/chloroform extraction with final RNA purification by solid-phase extraction on a glass-fiber filter. The residual genomic DNA was removed according to the manufacturer's instructions for DNase treatment using the DNA-free™ reagents provided in the kit. RNA quantity was determined by absorbance at 260 nm with a NanoDrop 1000 for all samples. RNA quality was spot-checked using a 2100 Bioanalyzer (Agilent 2100 expert software version B.02.05.SI360, Agilent, Palo Alto, CA). The genes used to normalize samples run on the custom monkey TLDA were *PGK1 *and *ZNF592*. At least one post-dose sample collected within the first day after dosing was tested for each of the three treated monkeys. However, some samples from some time-points did not yield RNA of sufficient quantity or quality for testing, and therefore there were less than 3 per group at some time points. Samples were stored at -80°C pending cDNA synthesis. 1800 ng of total RNA per sample was converted to cDNA.

With the exception of *IL2*, which was measured in a separate TaqMan^® ^assay (see below), TaqMan^® ^assays for genes with a known association with cytokine storm syndrome and/or an *ex vivo *rhIL21-dependent blood response in cynomolgus monkeys were measured using a custom TLDA for monkey studies as described previously (Arai *et al*. Journal of Translational Medicine, in press). Assays to measure the transcriptional levels of the following genes were included on the TLDA: *CD19, CSF1, GZMB, ICOS, IFNγ, IL2, IL10, IL21R, IL2RA, IL6, IL7, IL8, PRF1, STAT3, TBX21, TNF, CSF2, IL12B*. Monkey *IL2 *was assayed independently using ABI assay Rh02621714_m1 and 7500 Fast Real-Time PCR System and TaqMan^® ^Fast Universal PCR Master Mix (2X) Protocol. Levels of RNA were determined as described above. CT values >36 were considered unreliable and were excluded from analysis.

## Results

### Breadth of search for Ab-01-dependent activation signals

The effects of cross-linked Ab-01 on levels of RNA expression in human PBMCs were tested for the 96 genes on the Human Immune TLDA and on levels of secretion of 10 cytokines associated with cytokine storm and/or pro-inflammatory cascade. Binding of all IgG reagents to wells was confirmed by ELISA performed after cell harvest (data not shown). Negative controls included media alone and IgG_1_TM.

Two additional control Ig reagents were evaluated on PBMCs from two of the donors. IL21 stimulation served as the positive control for activation through IL21R. Cross-linked anti-CD28 Ab served as a positive control for activation of cytokine storm-associated genes by a cross-linked antibody. Summaries of the conditions and time points tested on PBMCs from each of 10 donors are listed in Tables 1 and 2. In an extensive series of experiments with cross-linked anti-CD28, the most robust responses were observed at 20 hours. In total, 675 RNA samples were assayed on 180 TLDA cards (more than 17,000 RNA measurements) and 13,000 MSD cytokine measurements were taken. Data are presented below from the 20 hour time-point for cytokine secretion, and from the 4 hour and 20 hour time points for RNA measurement because the most robust signals were observed in the positive controls at these time points.

**Table 1:** Summary of assays performed

		Daudi	D1	D2-D3	D4-D5	D6-D10	D11-D15
Coating	wet	-/+	+/+	-/-	-/-	-/-	-/-
	
	dry	-/+	+/+	+/+	+/+	+/+	+/+
	
	anti-IgG	-/+	+/+	+/+	+/+	+/+	-/-

Positive controls	rhIL21	-/+	+/+	+/+	+/+	+/+	+/+
	
	Anti-CD28	-/-	-/-	-/-	-/-	+/+	+/+

Negative controls	IgG_1_TM	-/+	+/+	+/+	+/+	+/+	+/+
	
	IgG_1_/Fc	-/-	-/-	+/+	-/-	-/-	-/-

Testing antibody	Ab-01 10 μg/well	-/-	-/-	-/-	-/-	+/+	-/-
	
	Ab-01 <10 μg/well	-/+	+/+	+/+	+/+	+/+	-/-

Assay performed on PBMCs from each of 15 healthy human donors. D indicates "donor". + indicates that assay was performed. - indicates that assay was not performed. Left of/indicates RNA. Right of/indicates protein.

**Table 2:** List of time points surveyed

	Assay Format	Time	Daudi	D1	D2 - D3	D4 - D5	D6 - D10
RNA	96 Gene Human	4 h	-	+	+	+	+
		
	Immune Card	20 h	-	+	+	+	+

Protein	MS6000 Human	4 h	+	-	+	+	+
		
	TH1/TH2 10-Plex	20 h	+	+	+	+	+
		
		48 h	+	+	-	-	-
		
		72 h	+	+	-	-	-

### Characterization of positive control responses

**Figure 1:**
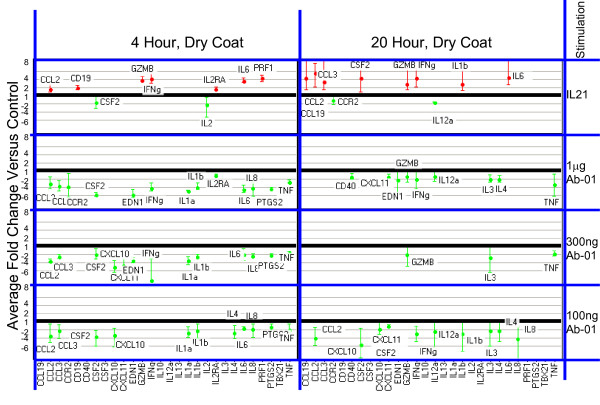
**RNA expression levels relative to negative control in IL21-responsive genes**. **Dry coated conditions.** RNA expression in cells cultured under indicated conditions. Mean fold changes and 95% confidence limits with Ab-01 relative to IgG_1_TM control (from 5 donors tested at concentrations less than 10 μg/well) are shown for genes where the absolute fold-change >1.5 and the 95% confidence limit excluded no change. For the IL21 tests, results are shown as average fold change relative to media control for all tests where the absolute fold-change >1.5 and the 95% confidence limit excluded no change. Confidence intervals were calculated in log-space and back-transformed and are represented by the error bars. Increases are shown in red, and decreases in green. The value 1 on the Y axis represents no change relative to control.

**Figure 2:**
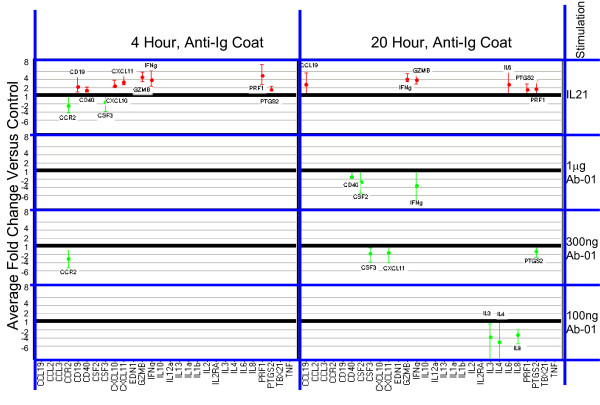
**RNA expression levels relative to negative control in IL21-responsive genes**. ** Anti-Ig coated conditions. **RNA expression in cells cultured under indicated conditions. Mean fold changes and 95% confidence limits with Ab-01 relative to IgG_1_TM control (from 5 donors tested at concentrations less than 10 μg/well) are shown for genes where the absolute fold-change >1.5 and the 95% confidence limit excluded no change. For the IL21 tests, results are shown as average fold change relative to media control for all tests where the absolute fold-change >1.5 and the 95% confidence limit excluded no change. Confidence intervals were calculated in log-space and back-transformed and are represented by the error bars. Increases are shown in red, and decreases in green. The value 1 on the Y axis represents no change relative to control.

To ensure that activation signals delivered through IL21R (the target of Ab-01) were detectable under the culture conditions used, rhIL21 was used as a positive control. Responses were detected by measuring the effects of *in vitro *stimulation with rhIL21 on both RNA and secreted cytokine levels in human PBMCs. Of the 96 genes tested for RNA expression, 21 gave a positive response to IL21 (at 95% confidence level) in at least one of the conditions tested (two time points and two plating conditions per time point). Results are presented in Figure 1 and 2. The most robust IL21-dependent changes were observed for *IFNγ, GZMB, PRF1 *and *IL6*. Significant rhIL21-dependent elevation of *IFNγ *RNA was observed under all conditions tested.

**Figure 3:**
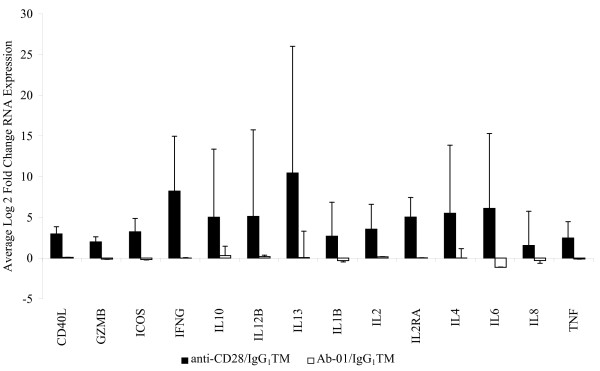
**Effect of cross-linked Ab-01 and anti-CD28 on RNA expression levels**. Data from 5 donors tested at 10 μg/well. Levels in the Ab-01 and anti-CD28 groups are expressed as fold change relative to IgG_1_TM control, +/- S.D.

**Figure 4:**
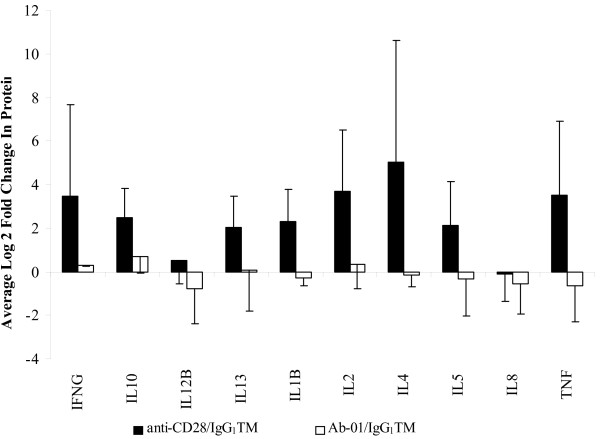
**Effect of cross-linked Ab-01 and anti-CD28 on cytokine secretion**. Data from 5 donors tested at 10 μg/well. Levels in the Ab-01 and anti-CD28 groups are expressed as fold change relative to IgG_1_TM control +/- S.D at 20 hours.

To confirm that the *in vitro *antibody cross-linking protocols reported by Stebbings *et al. *[14] induced cell activation in our hands, we tested the effects of *in vitro *cross-linked anti-CD28. Compared to IgG_1_TM control, cross-linked anti-CD28 induced large increases in RNA expression, and, consistent with the report of Stebbins *et al. *[14] also induced robust secretion of cytokine storm-associated proteins (Figures 3 and 4 respectively).

### Cross-linked Ab-01 does not induce detectable increases in either anti-CD28-responsive or IL21-responsive genes

A systematic comparison of results obtained with cross-linked Ab-01 and cross-linked control IgG_1_TM was conducted. Figure 1 and 2 summarizes the effects of Ab-01 at concentrations ranging from 0.1 to 1 μg/well on RNA expression levels of rhIL21-responsive genes. Results for anti-CD28 responsive genes at the 10 μg/well concentration of Ab-01 are shown in Figures 3 and 4. No significant increases in RNA expression (Figures 1, 2 and 3) or protein secretion (Figure 4) were observed in cells cultured in wells coated with cross-linked Ab-01.

There were numerous examples where expression level in the Ab-01 group was significantly (95% confidence level) lower than in the control IgG_1_TM group (Figure 1 and 2). When levels in IgG_1_TM and media alone were compared, levels in the IgG_1_TM cultures were significantly higher for a number of genes, indicating IgG_1_TM-dependent activation. To examine whether this activation was attributable to characteristics specific to that particular control reagent, two other cross-linked Ig control reagents were tested. Both of these reagents - human IgG_1 _wild type (which shares all characteristics with IgG_1_TM except the three mutations in the constant region), and purified human-Fc - induced similar increases over media control (data not shown).

### Analysis to probe for agonistic signal in each individual donor

In addition to the analyses presented in Figures 1, 2, 3 and 4 showing that the group stimulated with cross-linked Ab-01 did not differ significantly from the control IgG_1_TM-stimulated group, we also conducted an analysis to determine if the RNA levels of any gene in any individual donor was significantly higher (> 3 × SD and more than 1.5-fold above average of control) with Ab-01 than the range of increases over media control observed across donors for the control IgG_1_TM stimulated group.

This analysis was conducted to address concerns that any Ab-01-mediated increases that may occur in one or more donors would not be identified as significant when all donors were analyzed as a group. With a single exception described below, the results indicate that, among the 10 donors tested under various conditions with Ab-01, no donor gave an Ab-01 response for any gene that significantly exceeded the range observed for the control IgG_1_TM group. The single exception was 3.2-fold elevation above the average of control IgG_1_TM that was observed in *IL2RA *RNA at 20 hours in one donor, but not at the 4 hour time point (the optimal time point for that biomarker of IL21-mediated activation), and only at the 0.3 μg/well concentration.

### Comparison of Ab-01 binding to human and monkey cells

As assessed by FACS analysis, saturating staining levels of Ab-01 and % T and B cells stained were comparable (see Additional file 1, Figure S1), but more Ab-01 (0.009 μg/ml versus 0.006 μg/ml) was required for 50% staining saturation of cynomolgus monkey B cells than of human B cells. (This observed difference could indicate that efficacy in humans might be achieved at a lower dose than that needed for efficacy in monkey. However, for the purposes of the findings presented here, we note that, the *in vivo *Ab-01 levels achieved in monkeys and targeted in humans are at least 3 logs higher than the ng/ml range required for staining saturation *in vitro*. The difference between species in Ab-01 required for saturation staining therefore occurs within a concentration window unlikely to be relevant to *in vivo *studies.)

### Similar expression levels of rhIL21-responsive and cytokine storm-associated genes in monkeys before and after *in vivo *administration of Ab-01

**Figure 5:**
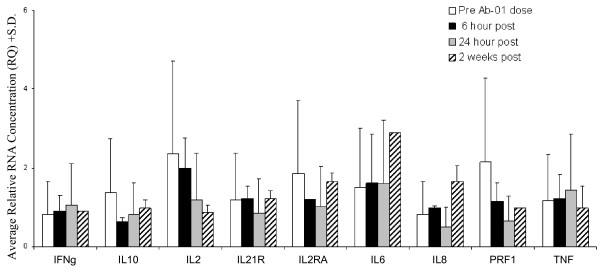
**Gene expression levels in blood of cynomolgus monkeys before and after IV administration of Ab-01**. Genes tested were selected based on known association with cytokine storm and/or reponsiveness of cynomolgus blood cells to *ex vivo *stimulation with rhIL21. Results from control untreated monkeys were comparable to Ab-01-treated monkeys. No signficant changes over pre-dose levels were observed, and no significant differences between control and treated monkeys were observed. Details of sampling by monkey is given in Additional file 2, Table S1.

Blood cell RNA expression levels of genes known to be associated with cytokine storm and/or IL21 response in cynomolgus monkeys were compared in Ab-01-treated and untreated animals. Consistent with the absence of symptoms of immunotoxicity in these animals (data not shown), post-dose gene expression levels were not significantly different from levels in control monkeys or from pre-dose levels in the two monkeys for which pre-dose samples were available (Figure 5). For the third monkey (for which no pre-dose sample was available) post-dose levels were similar to the levels in all other study samples. In experiments done in an independent set of monkeys, we investigated the changes in expression levels induced in blood samples by *ex vivo *stimulation with the polyclonal activators, the bacterial endotoxin LPS and the T cell mitogen PHA. Large increases in expre ssion levels were induced in monkey blood cell by these cell activators. For example, LPS induced a 40 fold increase in *TNF *and a 64 fold increase in *IL1β *expression. These data confirmed that the procedures used were capable of detecting changes in the expression levels of monkey genes, and also established that, relative to levels in activated cells, the levels of expression of these genes was low in control, pre-dose and post-dose samples.

## Discussion

For the study reported here, our goals were to conduct a broad search for an activation signal delivered *in vitro *by cross-linked Ab-01, an antagonist antibody to IL21R, and to assess whether *in vivo *dosing of the safety study species with Ab-01 resulted in gene activation. To assess the effects of *in vitro *cross-linked Ab-01, we used the protocols developed for the TGN1412 studies [14], but broadened the search to include additional protein measurements. We also measured RNA expression levels of a large set of pro-inflammatory mediators by TaqMan^® ^PCR, and in this way further expanded and greatly increased the sensitivity of the search. Inclusion of time points both preceding and following those tested in the TGN1412 study also broadened the search. Of the four plating conditions described by Stebbins *et al.*[14] we tested three, omitting the protocol for cross-linking via binding to human umbilical cord vein endothelial cells. The decision to omit this condition was made based on the report of more robust positive TGN1412-dependent activation reported with two of the three protocols that we did select.

With the background of the TGN1412 experience [15-19], concern exists that soluble antibodies, particularly those directed against immuno-modulatory cell-surface receptors, though devoid of *in vitro *agonistic properties may take on agonistic activities *in vivo*. In the case of TGN1412, it has been shown that the cytokine storm-associated genes activated by *in vivo *administration can also be activated in human PBMCs by cross-linked TGN1412 *in vitro *[14]. With the precedent of this study, we have gone to great lengths to ensure that a candidate therapeutic antibody against IL21R did not induce activation significantly above control when cross-linked *in vitro*.

We detected no effects on the expression levels of cytokine storm-associated genes following *in vivo *administration of Ab-01 to cynomolgus monkeys. The objective of this experiment was to test whether any presentation of Ab-01 *in vivo *could trigger activation of genes through cross-linked or other types of engagement of IL21R. It is must be noted, however, that no ill effects were reported following *in vivo *administration of TGN1412 to the non-human primate safety study species in those studies. It is therefore clear that any extrapolation between the results of *in vivo *Ab-01 studies in monkeys to an expectation of what might be expected upon treatment of humans with Ab-01 should be contingent upon meeting the following three conditions. First, a demonstration that Ab-01 has the desired and expected biological activity in this species is required. We have met this condition by showing that Ab-01 blocks IL21-mediated activation in monkeys [13], (Arai *et al*., Journal Of Translational Medicine, in press). Secondly, comparable levels of Ab-01 should be shown to bind to human and cynomolgus cells. We have met this condition by showing by FACS analysis that a given concentration of Ab-01 resulted in comparable levels of target engagement in humans and cynomolgus monkeys (see Additional file 1, Figure S1). Thirdly, comparable biological activity of Ab-01 should be demonstrated on human and monkey cells. We have met this condition by determining that the IC50 in cynomolgus monkey is only about 3.5-fold higher than the IC50 in human (Arai and LaVallie, unpublished data). Therefore, the lack of effect on gene expression of *in vivo *Ab-01 dosing of cynomolgus monkeys reflects absence of immunotoxicity in the presence of biological activity with target engagement at levels comparable to those in humans.

At the initiation of our study, we believed that our experimental plan favoured detection of a forced agonism signal. Our plan was to first identify such a signal and then use the signal as a biomarker of *in vivo *agonism in the safety study species. As reported here this plan was foiled by our failure to identify any Ab-01-dependent agonistic signal. We observed significant activation at both the RNA and protein level using three different control Ig reagents, and this finding is consistent with the many reports on signal transduction by cross-linked IgG [24-27]. Of note was the finding that a number of the genes significantly changed by rhIL21 were often observed to change less with Ab-01 than with control IgG_1_TM (Figure 3). These results suggest a negative effect on FcR-mediated signal transduction by antagonistic engagement of IL21R. These results are consistent with a hypothesis of reduced FcR-mediated pro-inflammatory cascades under conditions of IL21 pathway blockade. Reduction of immune complex-associated disease processes could be clinically beneficial in a number of diseases, including, for example, lupus nephritis. Further investigation of this possibility is contemplated.

The relevance of the *in vitro *cross-linked assays to the *in vivo *immunotoxicity observed with TGN1412 in the clinic is inferred from two lines of evidence: *in vitro *cross-linked TGN1412 induced activation in humans but did not do so in monkeys, and *in vivo *TGN1412 induced immunotoxicity in humans and not in monkeys.

However, it is clear that detection of a signal induced by cross-linked IgG that is above media control levels does not, by itself, predict *in vivo *immunotoxicity. The three different control Fc positive Ig reagents we used all showed elevation over media control of genes associated with cytokine storm, yet many antibodies containing these same Fc regions have been used extensively in the clinic without associated immunotoxicity. The activation signals observed with the control IgG reagents were predictable from the well-characterized signal induction known to occur through cross-linked FcR [24-27]. The level of activation observed with FcR cross-linking by control IgG_1_TM, while statistically significant, was extremely low in comparison to signals observed with anti-CD28. We conclude that if there is an as-yet-undefined threshold of *in vitro *activation predictive of *in vivo *immunotoxicity, control IgG reagents are below that threshold, and our results show Ab-01 to be even further below the threshold. This rank of Ab-01 at the bottom of the list in terms of responses to immunoglobulin reagents in the *in vitro *cross-lining assay strongly supports the conclusion that the risk of Ab-01-related immunotoxicity is low.

## Conclusions

Using protocols demonstrated to induce expression of cytokine storm-associated genes with a known immunotoxic agonist antibody, we have conducted a systematic study of the ability of Ab-01, a candidate therapeutic antibody directed against IL21R, to induce an activation signal through IL21R. Activation signals were not observed despite more extensive tests and more sensitive assays than have been reported with a known immunotoxic agonist antibody. In addition we have demonstrated that this anti-IL21R antibody binds to cynomolgus monkey blood cells and blocks IL21-mediated signal transduction. Despite this demonstration of the desired biological activity in the safety study species, symptoms of immunotoxicity were not observed, and activation of cytokine storm-associated genes was not detected, in cynomolgus monkeys treated IV with the anti-IL21R antibody. These studies have therefore shown that this anti-IL21R does not display the known pre-clinical characteristics of an immunotoxic antibody.

## List of abbreviations

LPS: lipopolysaccharide; PHA: phytohemaggultinin; PBMC: peripheral blood mononuclear cells; IRB: Institutional Review Board; IgG_1_TM: Human IgG_1 _anti-tetanus triple mutant; (containing three mutations in Fc portion); FDR: false discovery rate; Ab-01: anit-IL21R; also known as ATR-107; RQ: relative quantification (of RNA).

## Competing interests

All authors were employees of Pfizer (formerly Wyeth) at the time this work was performed.

## Authors' contributions

YG designed and performed all the *in vitro *studies, and the gene and protein expression assays for both human and cynomolgus studies reported here, co-wrote the manuscript, and with AAH, FIW and MOT performed the data analysis. AAH and FWI were study statisticians, RCR and CC assisted YG with experiments, DY and LB co-led the Ab-01 team with accountability for lead selection, cell-based and *in vivo *activity assays, and project advancement to development stage. ERL worked on data analysis review and manuscript preparation, MR performed the FACS based assessment of Ab-01 saturated staining in human and monkey cells, TB was responsible for the *in vivo *portion of the studies in cynomolgus monkey, RP, GW, and MB had responsibilities related to the planning and review of studies evaluating the possibility of agonistic activity of Ab-01. MOT, with YG, designed all experiments reported here, coordinated cross-functional activities, reviewed all results, supervised and participated in data analyses, and co-wrote the manuscript. All authors have read and approved the final manuscript.

## Supplementary Material

Additional file 1 Staining of lymphocytes with Ab-01 saturated at similar antibody concentrations in human and cynomolgus monkey. Click here for file

Additional file 2 Evaluable samples from control and Ab-01-treated cynomolgus monkeys. Click here for file
