# Supplementary material for: Assessing agonistic potential of a candidate therapeutic anti-IL21R antibody
Source: J Transl Med. 2010 May 26;8:50. doi: 10.1186/1479-5876-8-50 (PMC2896924; doi:10.1186/1479-5876-8-50)
Supplement: Additional file 1 — Staining of lymphocytes with Ab-01 saturated at similar antibody concentrations in human and cynomolgus monkey. [file 1479-5876-8-50-S1.PDF]

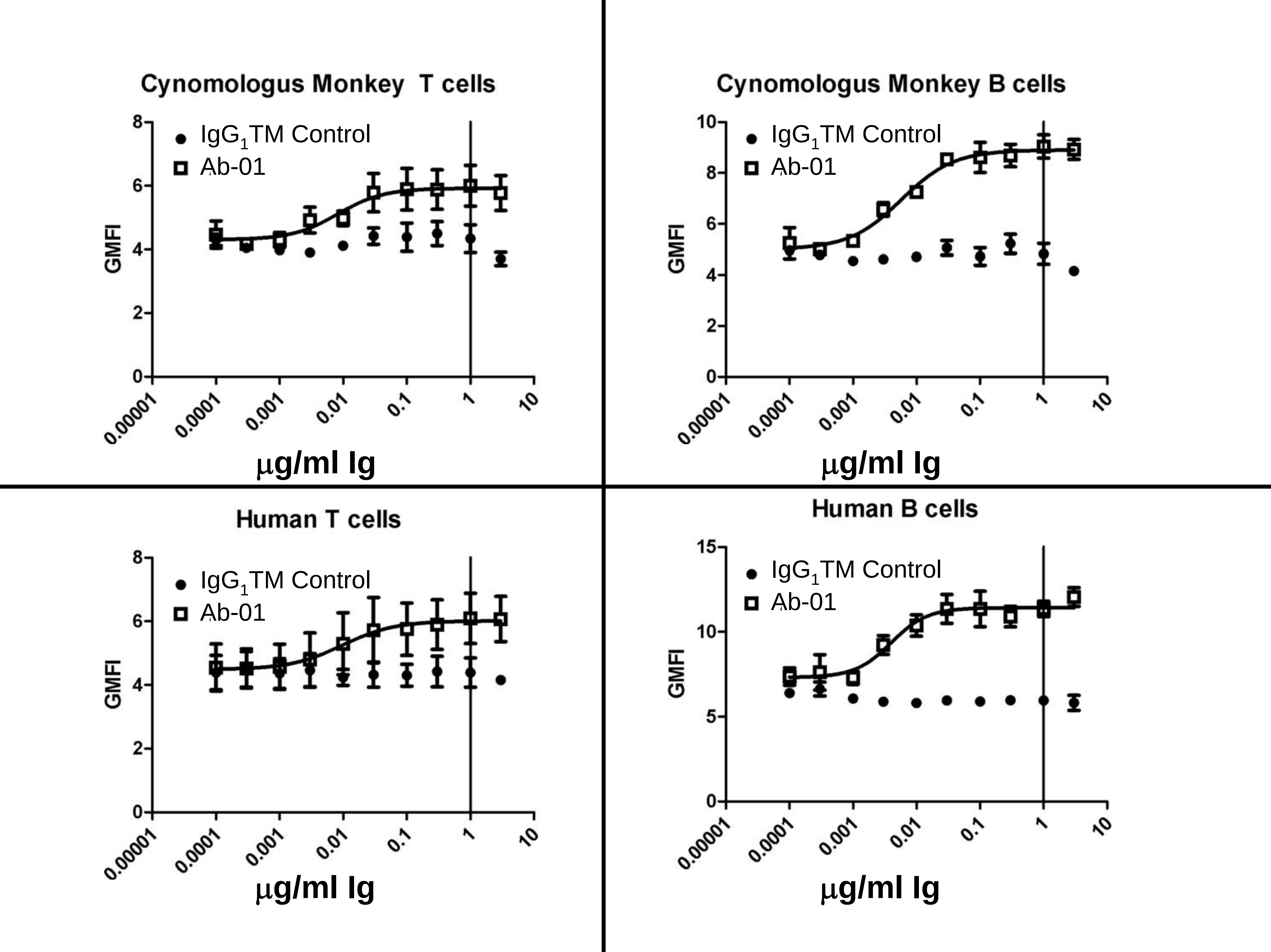
Cynomologus Monkey T cells
IgG1TM Control
Ab-01
GMFI
µg/ml Ig
Cynomologus Monkey B cells
IgG1TM Control
Ab-01
GMFI
µg/ml Ig
Human T cells
IgG1TM Control
Ab-01
GMFI
µg/ml Ig
Human B cells
IgG1TM Control
Ab-01
GMFI
µg/ml Ig

Additional file Figure 1 - Staining of lymphocytes with biotinylated Ab-01 is saturated at similar antibody concentrations in human and cynomolgus monkey. 100 μL aliquots of either human, or cynomologus monkey heparinized whole blood was incubated with the lineage markers, CD20- FITC, CD16-PE, CD3-APC, CD14-Alexa700 (BD-Biosciences, San Jose, CA), and the indicated concentration of biotinylated Ab-01 or control IgG_1TM for 30 minutes RT. Samples were washed in 2 mL with PBS containing 0.5% BSA, centrifuged at 300g for 7minutes at 4ºC. The supernatants were aspirated, and the pellets resuspended in 100 μL of cold PBS/0.5%BSA buffer containing 10 μg/mL streptavidin Alexa405 (Life Technologies, Carlsbad, CA). After 30 minutes, 2mL of 1x BD Facs/Lyse (BD-Biosciences, San Jose, CA) was added and the preps were incubated at room temperature for 10 minutes to lyse the red blood cells. The cells were centrifuged as before, the supernatants aspirated and the cells resuspended in 300 μL of cold PBS/BSA and acquired on an LSRII flow cytometer (BD Biosciences, San Jose, CA). Data are expressed as Geometric Mean Fluorescence Intensity (GMFI).
