# Supplementary material for: Assessing agonistic potential of a candidate therapeutic anti-IL21R antibody
Source: J Transl Med. 2010 May 26;8:50. doi: 10.1186/1479-5876-8-50 (PMC2896924; doi:10.1186/1479-5876-8-50)
Supplement: Additional file 2 — Evaluable samples from control and Ab-01-treated cynomolgus monkeys. [file 1479-5876-8-50-S2.PDF]

Additional file Table S1: Evaluable samples from control and Ab-01-treated cynomolgus monkeys

Animal	Cohort	Pre-dose	6 hours	24 hours	2 weeks
**1**	Control			+	
**2**	Control	+	+	+	+
**3**	Control	+		+	+
**4**	Ab-01	+	+	+	
**5**	Ab-01	+	+		+
**6**	Ab-01			+	
